# circ_0029463 promotes osteoclast differentiation by mediating miR-134-5p/Rab27a axis

**DOI:** 10.1186/s13018-024-04610-5

**Published:** 2024-02-07

**Authors:** Lian Tang, Lin Yuan, Jiyuan Yan, Jianhua Ge, Zhi Lian, Zhong Li

**Affiliations:** 1 Department of Orthopedics, Affiliated Hospital of Southwest Medical University, No. 25 Taiping Street, Jiangyang District, Luzhou, 646000 Sichuan People’s Republic of China; 2 Department of Clinical Skills Center, Affiliated Hospital of Southwest Medical University, Luzhou, 646000 Sichuan People’s Republic of China; 3 Southwest Medical University, Luzhou, 646000 Sichuan People’s Republic of China; 4 Stem Cell Immunity and Regeneration Key Laboratory of Luzhou, Affiliated Hospital of Southwest Medical University, Luzhou, 646000 Sichuan People’s Republic of China

**Keywords:** circ_0029463, miR-134-5p, Rab27a, Osteoclast differentiation

## Abstract

**Objective:**

Osteoporosis is the imbalance in bone homeostasis between osteoblasts and osteoclasts. In this study, we investigated the effects of the circ_0029463/miR-134-5p/Rab27a axis on RANKL-induced osteoclast differentiation.

**Methods:**

RT-qPCR and western blotting were used to detect the expression of circ_0029463, miR-134-5p, and Rab27a in tissues from patients with osteoporosis and in RANKL-induced osteoclasts. Osteoclast differentiation was verified by TRAP staining. Osteoclast biomarkers, including NFATc1, TRAP, and CTSK, were measured. The target and regulatory relationships between circ_0029463, miR-134-5p, and the Rab27a axis were verified using RIP, dual-luciferase reporter gene, and RNA pull-down assays.

**Results:**

Elevated expression of circ_0029463 and Rab27a and decreased miR-134-5p expression were observed in the tissues of patients with osteoporosis, and a similar expression pattern was observed in RANKL-induced osteoclasts. Suppression of circ_0029463 expression or miR-134-5p overexpression curbed RANKL-induced osteoclast differentiation, whereas such an effect was abolished by Rab27 overexpression. circ_0029463 sponges miR-134-5p to induce Rab27a expression.

**Conclusion:**

circ_0029463 sponges miR-134-5p to abolish its suppressive effect of miR-134-5p on Rab27a expression, thereby promoting osteoclast differentiation.

## Introduction

Osteoporosis is a well-known systemic skeletal disorder characterized by decreased bone strength due to deteriorated bone microarchitecture, resulting in increased vulnerability to fractures and increased bone fragility [1]. During bone remodeling, multiple molecules are involved in the coordination of bone cells such as osteoblasts and osteoclasts; knowledge of the underlying bone cell function contributes to understanding the biology of osteoporosis and seeking therapeutics by targeting key molecules [2]. Osteoclasts are key regulators of skeletal disorders such as osteoporosis and may mediate osteoblast differentiation, hematopoietic stem cell niche, T cell activation, and skewing [3]. Osteoclasts are primary bone-resorptive cells that contribute to bone remodeling. Defects in osteoclasts cause an imbalance in bone remodeling, leading to pathological disorders, including osseous metastasis, osteoporosis, and inflammatory bone erosion [4]. Hence, identifying molecules such as non-coding RNAs that regulate the activities of osteoclasts will aid in the understanding of bone pathophysiology.

Non-coding RNAs (ncRNAs), including microRNAs (miRNAs), long non-coding RNAs, and circular RNAs (circRNAs), are small RNA molecules that are not translated into proteins but have the ability to modulate the translation of target mRNA [5, 6]. The role of ncRNAs in musculoskeletal conditions has been previously [7–11]. CircRNAs were found to mediate the expression of genes related to osteoclast and osteoblast differentiation [12], showing potential as biomarkers for osteoporosis [13]. Furthermore, high-throughput RNA sequencing and circRNA microarray analyses have documented dysregulation of circRNAs in osteoporosis [14]. Another study using microarray analysis identified 237 differentially expressed circRNAs (162 upregulated and 95 downregulated circRNAs) between osteoporosis and control groups [15]. The circular RNA circ_0029463 is encoded by *EP400*. Ep400 is composed of 1370 bp, and the spliced mature circRNA is also composed of 1370 bp [16]. CircRNA-Ep400 is localized in the cytoplasm and is derived from the circulating exons of the Ep400 gene, which is located on chr5:111184415–111185785 [16]. In the GEO dataset GSE161361, circ_0029463 is upregulated in patients with osteoporosis. However, based on current understanding, less is known regarding how circ_0029463 regulates osteoporosis progression.

Data proved that CircRNAs may participate in the regulation of physiological or pathological processes by mediating transcription and protein and miRNA functions via different mechanisms in specific tissues [17]. Recent studies have revealed vital roles of circRNA-miRNA-mRNA networks in the progression of osteoporosis [18, 19]. Targeting these circRNAs or miRNAs may be a promising anti-osteoporotic approach. Our bioinformatics analysis further revealed a binding relationship between circ_0029463 and miR-134-5p, as well as between miR-134-5p and Rab27a, indicating a potential circ_0029463-miR-134-5p-Rab27a network. A recent study reported that miR-134-5p suppresses osteoclastogenesis and osteoclast differentiation in bone marrow-derived macrophages (BMMs) [20]. Rab27a has also been suggested as a promoter of osteogenesis [21]. The binding relationship between miR-134-5p and Rab27a has been reported on ovarian cancer cells [22]; however, whether miR-134-5p can mediate Rab27a expression in osteoclasts remains to be determined. Therefore, it was necessary to validate whether the circ_0029463-miR-134-5p-Rab27a network could be implicated in osteoclast formation and differentiation. In this study, we aimed to ascertain the regulatory role of this network in osteoclast differentiation to gain a new mechanistic understanding of osteoclastogenesis and provide a theoretical basis for the development of molecular targets.

## Materials and methods

### Ethical statement

The clinical and animal experimental designs were approved by the Ethics Committee of our hospital (Approval no. 20221028-017). The clinical experiments were conducted in accordance with the *Declaration of Helsinki* and the written informed consent from all the included patients was collected from tissue collection. Animal experiments were performed in accordance with the experimental guidelines of our hospital.

### Collection of clinical tissues

Postmenopausal patients (*n* = 40) admitted to our hospital between January 2021 and June 2022, who were scheduled to undergo total hip arthroplasty due to hip osteoarthritis, were recruited based on the following inclusion criteria: (1) 50–70 years old; (2) natural menopause, menopause for more than 1 year. Patients who met the following criteria were excluded: (1) serious cardio- or cerebrovascular diseases, diabetes mellitus, and liver or renal dysfunction; (2) combined with hyperthyroidism, rheumatoid arthritis, or other diseases that can cause secondary osteoporosis; (3) having a glucocorticoid hormone application record; and (4) taking medicines that may affect bone metabolism within the past 6 months. The femoral neck bone mineral density (BMD) on the operated side was preoperatively measured using a dual-energy X-ray scanner (Medilink, France) and was accordingly classified into the osteoporosis group (T-value < − 2.5, 20 cases) and normal bone mass group (T-value > − 1.0, 20 cases). The patients in the osteoporosis group were ranged from 55 to 72 years old with median age of 60.9 years old, body mass index (BMI) of 23.45 ± 2.45, femoral neck BMD of (0.72 ± 0.05) g/cm^2^, while patients in the normal BMD group was ranged from 52 to 67 years old with the median age of 58.8 years old, BMI of 24.25 ± 2.95 and femoral neck BMD of (1.04 ± 0.08) g/cm^2^. The cancellous bone of the femur was collected using aseptic bone cutting forceps during the total hip arthroplasty and transferred using in liquid nitrogen for storage in an − 80 °C fridge.

### RT-qPCR

Total RNA was extracted from cancellous bones and osteoclasts using TRIZOL (Invitrogen, Carlsbad, CA, USA). An RT kit (TaKaRa, Tokyo, Japan) or miRNA First Strand cDNA Synthesis (Tailing Reaction) kit (B532451-0020, Sangon, China) was used to obtain cDNA strictly based on the instructions for mRNA and miRNA, respectively. PCR was performed on a 7300 real-time PCR system (ABI, Foster City, CA, USA) using a SYBR Green Mix kit (Takara). Data were calculated based on 2^−ΔΔCt^ method [23] with GAPDH as the internal gene. The sequences used for PCR are listed in Table 1.

**Table 1:** Primer sequences

Name of primer	Sequences (5′–3′)
circ_0029463-F	TGGACCTGATGAAGCTGTACG
circ_0029463-R	ATCTCTGAGAGCCCTGCGAC
miR-134-5p-F	TATGTGACTGGTTGACCAGAGGGG
EP400-F	CCGACCCCTTTAAGAGGCAA
EP400-R	ACCCCTGGATGCTGGAAAAC
miR-134-5p-R	GTGCAGGGTCCGAGGT
Rab27a-F	CGGAATCCCCTATTTTGAAACC
Rab27a-R	CTTCTCCTTCTCCTCACTTAGC
NFATc1-F	GGTGCCTTTTGCGAGCAGTATC
NFATc1-R	CGTATGGACCAGAATGTGACGG
TRAP-F	CCGTGTTCCTACCCCCAATG
TRAP-R	GGTCTCCTGGAACCTCTTGT
CTSK-F	CTTCCAATACGTGCAGCAGA
CTSK-R	TTGCATCGATGGACACAGAG
U6-F	CTCGCTTCGGCAGCACA
U6-R	AACGCTTCACGAATTTGCGT
GAPDH-F	AGCCCAAGATGCCCTTCAGT
GAPDH-R	CCGTGTTCCTACCCCCAATG
miR-134-5p-F (Human)	GCAGTGTGACTGGTTGAC
miR-134-5p-R (Human)	GCAGTGTGACTGGTTGAC
U6-F (Human)	CTCGCTTCGGCAGCACA
U6-R (Human)	AACGCTTCACGAATTTGCGT
Rab27a-F (Human)	ACAGCGTTCTTCAGAGATGCTATGG
Rab27a-R (Human)	TCTGCGAGTGCTATGGCTTCCT
GAPDH-F (Human)	TCAAGAAGGTGGTGAAGCAGG
GAPDH-R (Human)	TCAAAGGTGGAGGAGTGGGT

*F* forward, *R* reverse

### Western blot

Osteoclasts were lysed using RIPA lysis buffer (Beyotime) to obtain proteins, which were further quantified using a bicinchoninic acid (BCA) kit (Beyotime). The mixed protein and loading buffer were heated in a boiling water bath for 3 min and subjected to electrophoresis at 80 and 120 V for 30 and 90 min, respectively. The membrane transferred was performed with current at 250 mA for 100 min, after that the membranes were washed in washing buffer for 1–2 min and treated with blocking buffer at 4 °C for overnight. The proteins were incubated with primary antibodies against Rab27a (ab55667, 1:1000, Abcam), CTSK (ab187647, 1:1000, Abcam), TRAP (ab52750, 1:1000, Abcam), NFATc1 (#4389, 1:1000, Cell Signaling Technology), and GAPDH (ab8226, 1:5000, Abcam) at room temperature for 1 h and then washed for 3 × 10 min. Secondary antibodies used were goat anti-rabbit IgG (ab6702, 1:5000; Abcam) and goat anti-rat IgG (ab6708, 1:5000; Abcam). After color development, the trips in the membranes were detected using a chemiluminescence imaging system (Bio-Rad, Hercules, CA, USA).

### Animals

Six SPF C57BL mice (6–8 weeks) from Hunan Slac Laboratory Animal Co., Ltd were kept in cages with temperature of 21–25 °C, humidity of 50–65% and a 12-h:12-h light–dark cycle. Food and water were accessible to all mice.

### Extraction and culture of BMMs

The mice were anesthetized and decapitated with bilateral ankles of the lower extremity, knee joint, and hip joints. The tibia and femur were placed in Petri dishes containing PBS and removed from the surface tissues. The marrow cavity was exposed after the bone tissues at the proximal end of the tibia and distal end of the femur were removed. Then a 1 ml syringe with PBS was used to wash the marrow cavity and the marrow collected was resuspended in α-MEM culture medium (SH30265.01, Hyclone, USA) at 1500 r/min for 4 min. After abandon the supernatant, the marrow was resuspended in complete culture medium containing 30 ng/ml M-CSF and incubated overnight in a 10 cm culture disk. On the next day, the culture medium was discarded, and the remaining cells were washed in PBS three times and cultured in complete culture medium for 2 days. Once the cells had a short shuttle-shape and the cell concentration reached 80–90%, they were digested with trypsin. The cells were counted and identified as BMMs.

### BMMs induced for osteoclast differentiation

BMMs at the concentration of 1 × 10^5^ cells/well were placed in a 24-well plate for overnight growth before induced for osteoclast differentiation using α-MEM complete culture medium + 50 ng/ml RANKL + 30 ng/ml M-CSF. The culture medium was replaced every 2 days. On day 4, cell fusion was observed and the cells presented with more than three nuclei. On day 6, the BMMs were round and pancake-like in shape with a clear boundary.

### Cell transfection

The vectors used for cell transfection were obtained from Hanbio (Shanghai, China) and included sh-circ_0029463 (circ_0029463 suppression), sh-NC, oe-WTAP (WTAP overexpression), oe-NC, in-miR-134-5p (miR-134-5p inhibitor), in-NC (inhibitor NC), miR-134-5p mimic, mimic NC, oe-Rab27a (Rab27a overexpression), and oe-NC. After 48 h of cell transfection, the viral titer in the transfected 293 T cells was measured using a p24 ELISA kit (Cell Biolabs, Inc., San Diego, USA). BMMs were transfected for 24 h and further cultured for 48 h before the stably transfected cell lines were screened using puromycin (P8230; Solarbio, Beijing, China). Transfection efficiency was validated before cell biological functions were assessed.

### Detection of osteogenesis differentiation ability of BMMs

On day 6 of osteogenic differentiation**,** the culture medium of the cells in the 24-well plate was removed, and the cells were washed with PBS before fixation with 4% polyformaldehyde for 10 min and TRAP staining using a TRAP staining kit (Sigma-Aldrich, St. Louis, MI, USA). TRAP-positive cells with more than three nuclei observed under a microscope (Olympus, Tokyo, Japan) were considered as osteoclasts. The relative size of osteoclasts = osteoclast size in each group/mean size of osteoclasts in the solvent control group.

### RNA immunoprecipitation (RIP) assay

Cells were treated with IP lysis buffer before incubated with the mixture of Pierce™ Protein A/G magnetic beads (Thermo Fisher Scientific, USA) in EZ-MagnaRIP kit (Millipore, MA, USA) with rabbit anti Ago2 (ab32381, abcam) or normal rabbit anti IgG (ab18877, abcam) at 4 °C for 4 h. The complex was eluted and purified using TRIzol reagent before quantification by RT-qPCR.

### Dual-luciferase gene reporter assay

StarBase (http://starbase.sysu.edu.cn/) predicted the binding sites of miR-134-5p with circ_0029463 and TargetScan (http://www.targetscan.org/vert_72/) predicted the binding sites of miR-134a-5p with Rab27a. The mutant and wild-type sequences (wt-circ_0029463, mut-circ_0029463, wt-Rab27a, and mut-Rab27a) were synthesized and inserted into PGL3-Promotor for co-transfection with the miR-134a-5p mimic (30 nM) or negative control into BMMs. The fluorescence intensity was detected using a dual-luciferase gene reporter assay kit (Promega, Madison, WI, USA) and a luminometer (Turner BioSystems, USA).

### RNA pull-down

Cell lysis isolated from BMMs was incubated with 100 ng biotin labeled NC-probe or miR-134-5p probe at 25 °C for 2 h. The complex was captured using streptavidin marked immunomagnetic beads at 25 °C for 1 h before digested with buffer which contains proteinase K at 25 °C for 1 h. The eluted complexes were further analyzed using qRT-PCR.

### Statistical analysis

Data were processed with GraphPad prism8 and expressed as mean ± standard deviation ($\overline{x}$ ± SD). Comparisons between two groups were analyzed using the *t* test, whereas comparisons among multiple groups were determined using one-way analysis of variance (ANOVA) and Tukey’s multiple comparison test. Statistical significance was set at *P* < 0.05.

## Results

### Elevated expression of circ_0029463 in both patients with osteoporosis and RANKL-induced osteoclasts

**Fig. 1:**
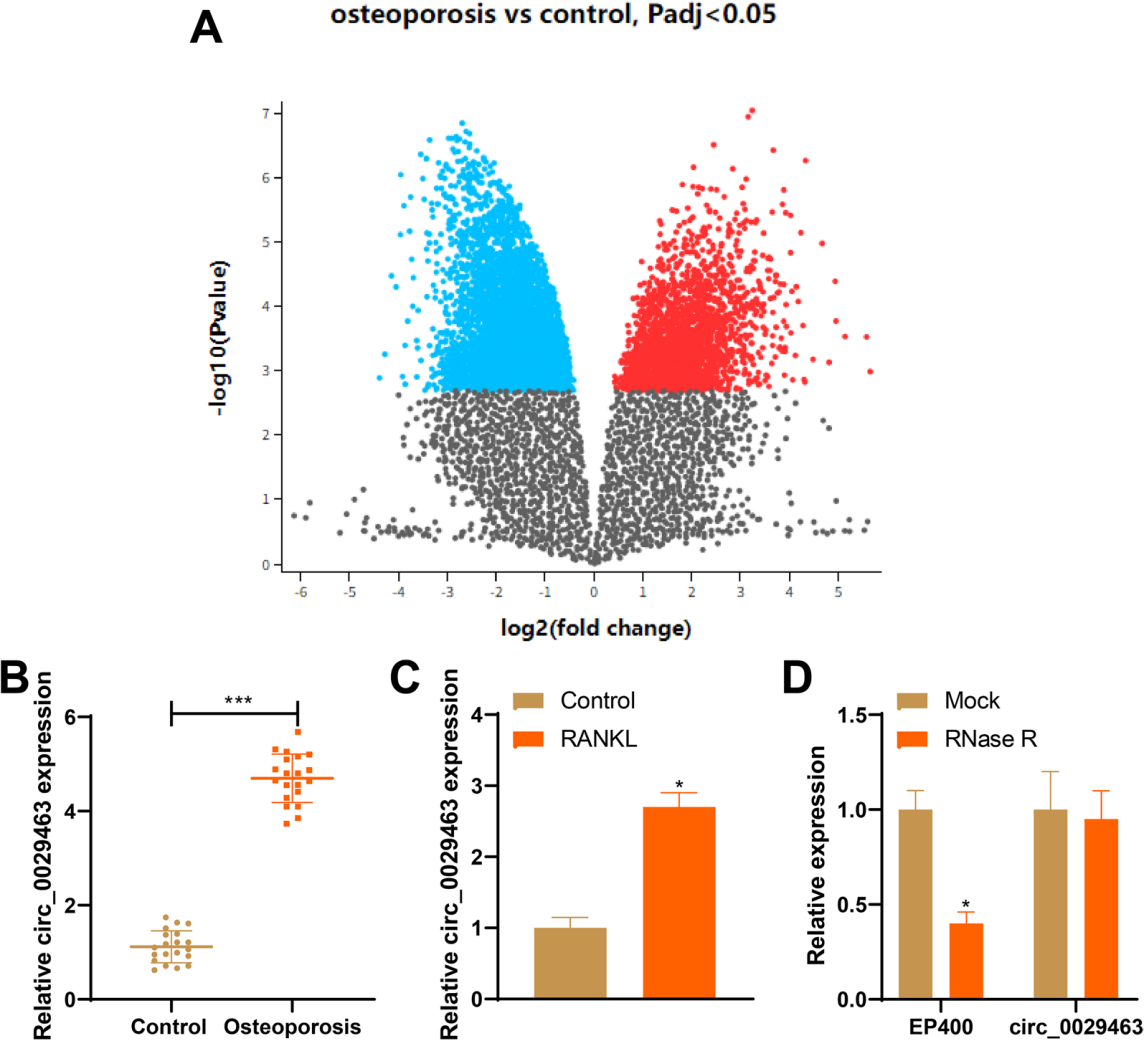
circ_0029463 is highly expressed in patients with osteoporosis and osteoclast. **A** circ_0029463 expression pattern in patients with osteoporosis based on data obtained from GEO database; **B**, **C** RT-qPCR detected circ_0029463 expression in patients with osteoporosis and osteoclasts; **D** RT-qPCR detected the stability of circ_0029463 after Rnase R treatment. Data are expressed as mean ± standard deviation, clinical experiments *N* = 20, cell experiments were repeated 3 times. *P < 0.05, when compared with Control or Mock group

Based on data obtained from the GEO database, circ_0029463 expression was elevated in patients with osteoporosis (Fig. 1A), which was consistent with the findings of the current study (Fig. 1B, *P < 0.05).

BMMs extracted from mice were treated with M-CSF and RANKL to induce osteoclast differentiation, and circ_0029463 expression was detected. The circ_0029463 expression was higher in RANKL group than that in the control group (Fig. 1C, *P < 0.05). Moreover, the stability of circ_0029463 expression was compared to that of the mock treatment. RNase R treatment suppressed EP400 expression instead of circ_0029463 expression (Fig. 1D, *P < 0.05), suggesting that the ring structure of circ_0029463.

### circ_0029463 knockdown suppresses osteoclast differentiation

**Fig. 2:**
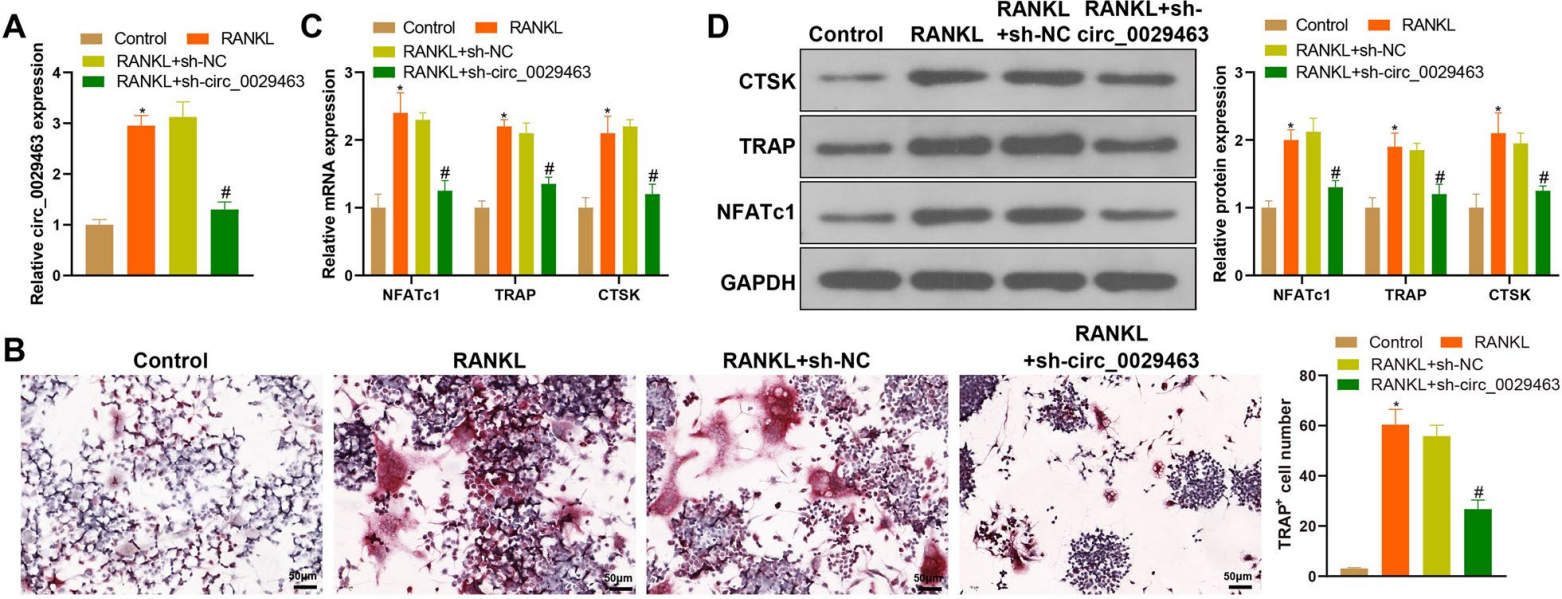
Osteoclast differentiation was inhibited in response to circ_0029463 knockdown. **A** circ_0029463 expression was detected using RT-qPCR after sh-circ_0029463 vector was transfected; **B** osteoclasts were observed after TRAP staining; **C**, **D** the biomarkers of osteoclasts, including NFATc1, TRAP and CTSK were detected using RT-qPCR and western blot. Data are expressed as mean ± standard deviation, cell experiments were repeated 3 times. *P < 0.05, when compared with Control group; ^#^*P* < 0.05, when compared with RANKL + sh-NC group

The sh-circ_0029463 vector was transfected into BMMs to knock down circ_0029463. First, transfection efficiency was verified by RT-qPCR, which showed that sh-circ_0029463 successfully suppressed circ_0029463 expression in BMMs (Fig. 2A, ^*#*^*P* < 0.05). TRAP staining and assays demonstrated increased cell number and size in the RANKL group compared with those in the control group but were inhibited in the RANKL + sh-circ_0029463 group when compared with the RANKL + sh-NC group (Fig. 2B, ^*#*^*P* < 0.05). The detection of osteoclast biomarkers by RT-qPCR and western blotting revealed elevated mRNA and protein expression of NFATc1, TRAP, and CTSK in the RANKL group, compared to the control group (Fig. 2C, D, ^*#*^*P* < 0.05), while those expressions were reversed in the RANKL + sh-circ_0029463 group (Fig. 2B, D, ^*#*^*P* < 0.05).

### circ_0029463 negatively regulates miR-134-5p to mediate osteoclast differentiation

**Fig. 3:**
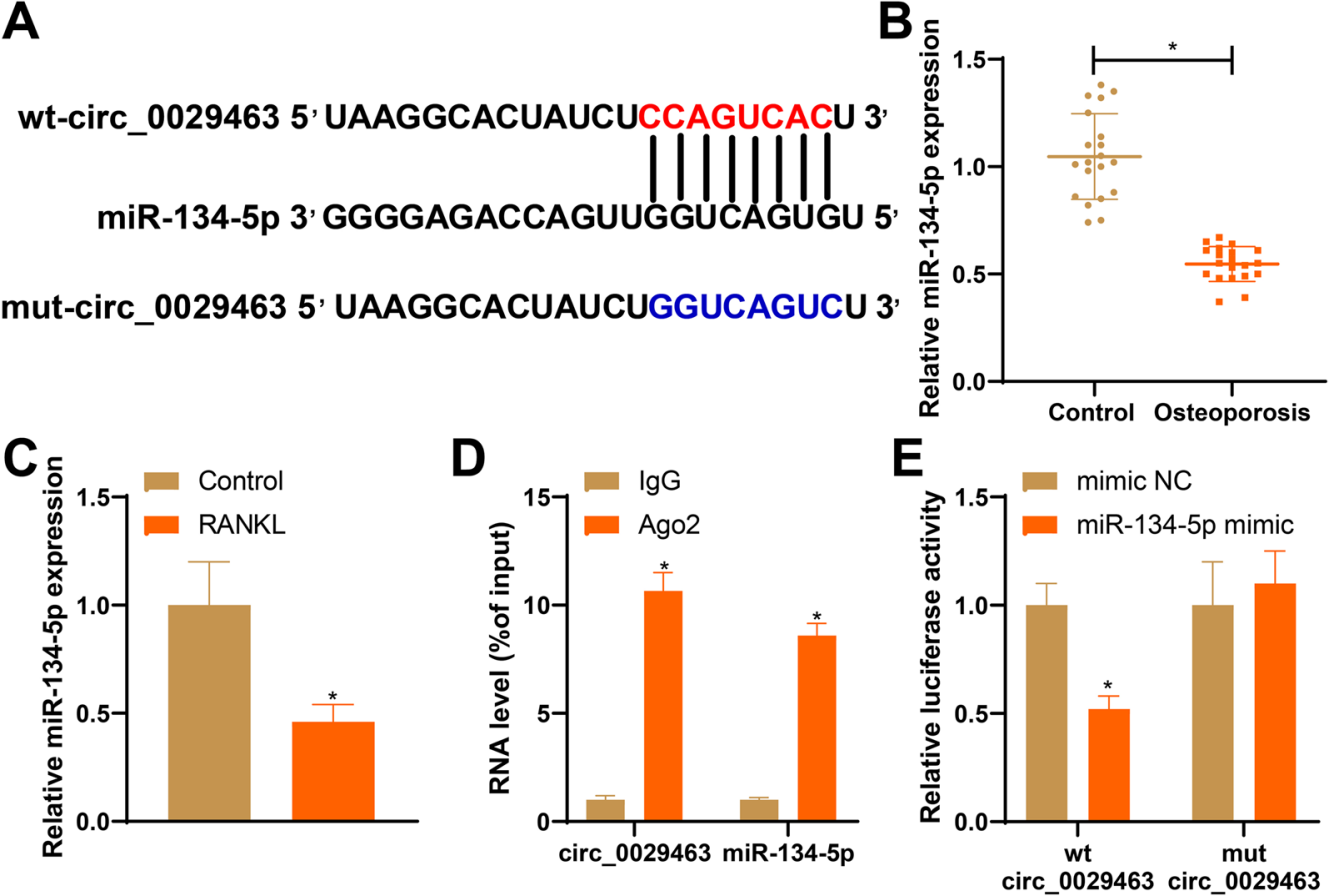
circ_0029463 negatively regulates miR-134-5p expression. **A** Binding sites of circ_0029463 with miR-134-5p; **B**, **C** RT-qPCR detected the expression of miR-134-5p in patients with osteoporosis and osteoclasts, respectively; **D**, **E** RIP and dual-luciferase reporter gene assay both verified the regulation of circ_0029463 on miR-134-5p. Data are expressed as mean ± standard deviation, cell experiments were repeated 3 times. *P < 0.05, when compared with Control, mimic NC or IgG group

The binding sites between circ_0029463 and miR-134-5p in StarBase are shown in Fig. 3A. RT-qPCR identified repressed miR-134-5p expression in the RANKL group and patients with osteoporosis compared to that in the control group (Fig. 3B, C, *P < 0.05). To verify the regulation of miR-134-5p in osteoclasts by circ_0029463, we examined its relationship using RIP and dual-luciferase reporter gene assays. RIP group showed substantially increased circ_0029463 and miR-134-5p expressions enriched by Ago2 were substantially boosted (Fig. 3D, *P < 0.05). Similar results were obtained by the dual-luciferase reporter gene assay, which showed that the miR-134-5p mimic could curb the fluorescence intensity of the wt-circ_0029463 group, instead of the mut-circ_0029463 group (Fig. 3E, *P < 0.05). These results suggested that circ_0029463 binds to miR-134-5p.

**Fig. 4:**
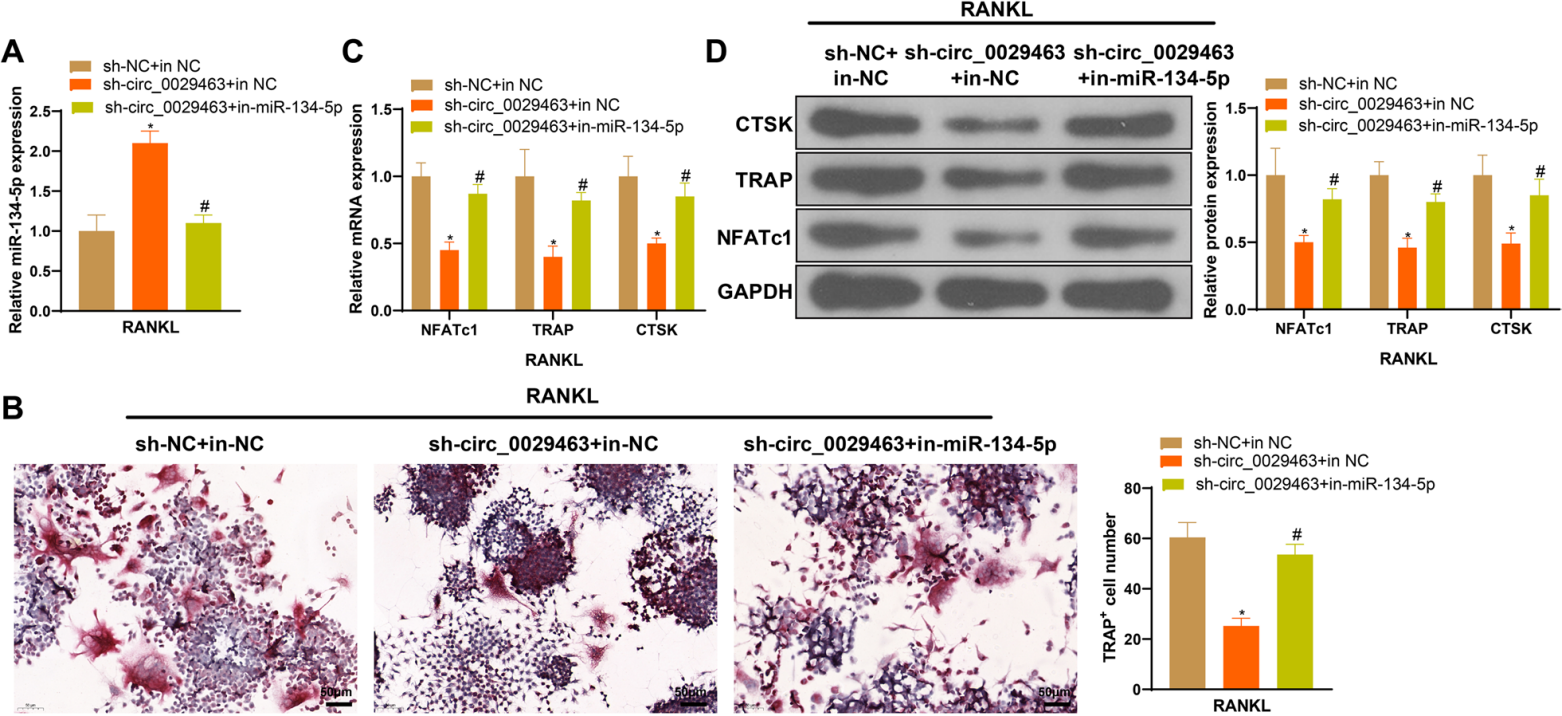
miR-134-5p suppression can reverse the effect of circ_0029463 on osteoclast differentiation. **A** miR-134-5 expression was detected using RT-qPCR; **B** osteoclasts were observed after TRAP staining; **C**, **D** the biomarkers of osteoclasts, including NFATc1, TRAP and CTSK were detected using RT-qPCR and western blot. Data are expressed as mean ± standard deviation, cell experiments were repeated 3 times. *P < 0.05, when compared with sh-NC + in-NC group; ^#^*P* < 0.05, when compared with sh-circ_0029463 + in-NC group

To further prove the regulation of circ_0029463 and miR-134-5p, we co-transfected sh-circ_0029463 and in-miR-134-5p into osteoclasts and found sh-circ_0029463 + in-NC group with elevated miR-134-5p expression compared to sh-NC + in-NC group (*P < 0.05), whereas miR-134-5p expression was inhibited in the sh-circ_0029463 + in-miR-134-5p group compared to that in the sh-circ_0029463 + in-NC group (Fig. 4A, *P < 0.05). Further evaluation of osteoclasts showed that in-miR-134-5p transfection abolished the suppressive effect of sh-circ_0029463 on osteoclast formation and differentiation (Fig. 4B–D, *P < 0.05).

### miR-134-5p negatively regulates Rab27a to mediate osteoclast differentiation

**Fig. 5:**
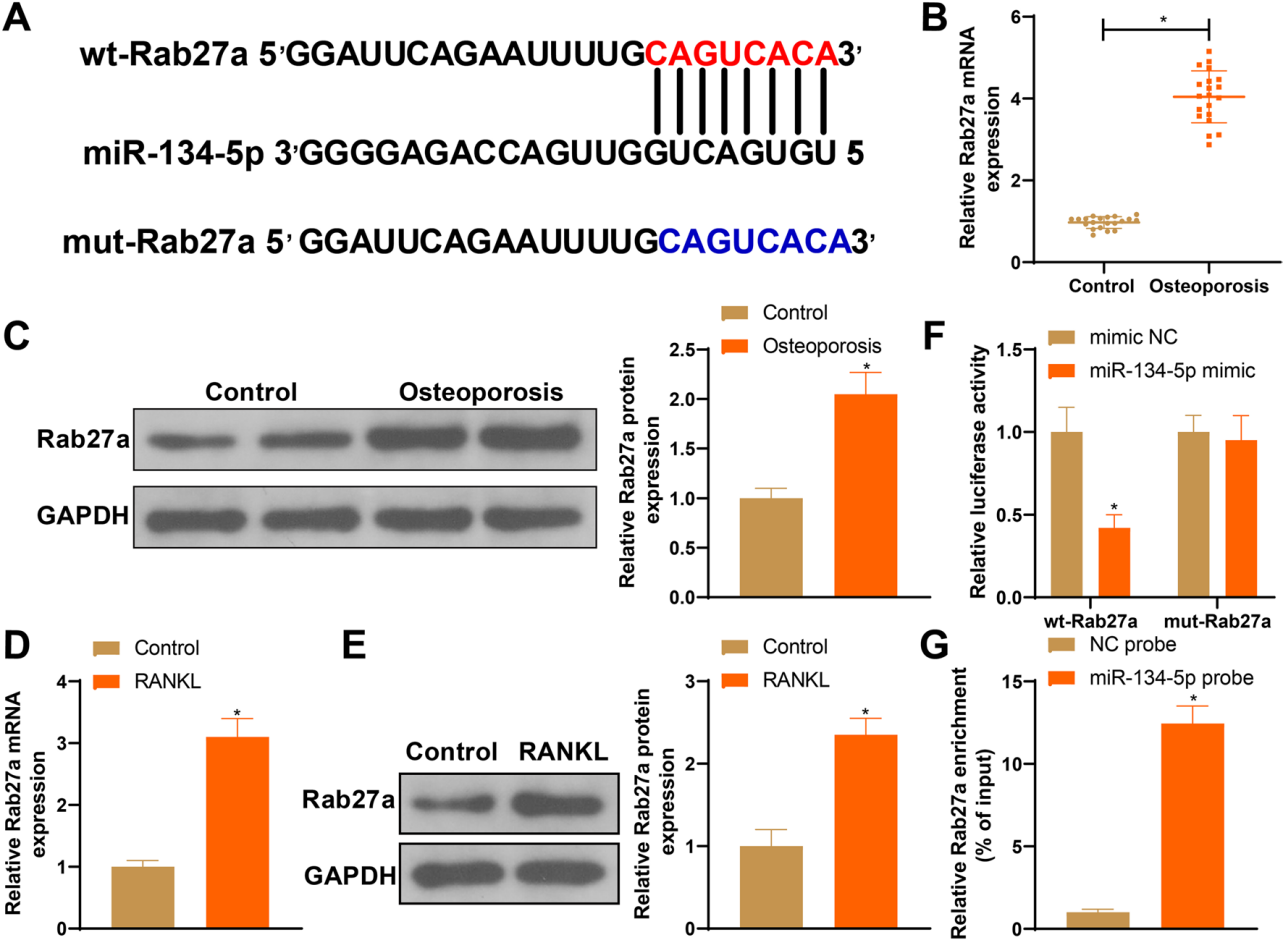
miR-134-5p negatively regulates Rab27a expression. **A** Binding sites between miR-134-5p and Rab27a; **B**–**E** Rab27a mRNA and protein expressions detected by RT-qPCR and western blot; **F**–**G** dual-luciferase reporter gene assay and RNA pull down verified the binding of miR-134-5p with Rab27a. Data are expressed as mean ± standard deviation, cell experiments were repeated 3 times. *P < 0.05, when compared with Control, mimic NC or NC probe group

The binding sites of miR-134-5p with Rab27a were also identified by bioinformatic analysis (Fig. 5A); therefore, we speculated that Rab27a may act as a downstream target of miR-134-5p during osteoclast differentiation. Elevated mRNA and protein expression of Rab27a detected in patients with osteoporosis and in osteoclasts confirmed our hypothesis (Fig. 5B–E, *P < 0.05). Dual-luciferase reporter gene and RNA pull-down assays verified the target relationship between miR-134-5p and Rab27a (Fig. 5F–G, *P < 0.05).

**Fig. 6:**
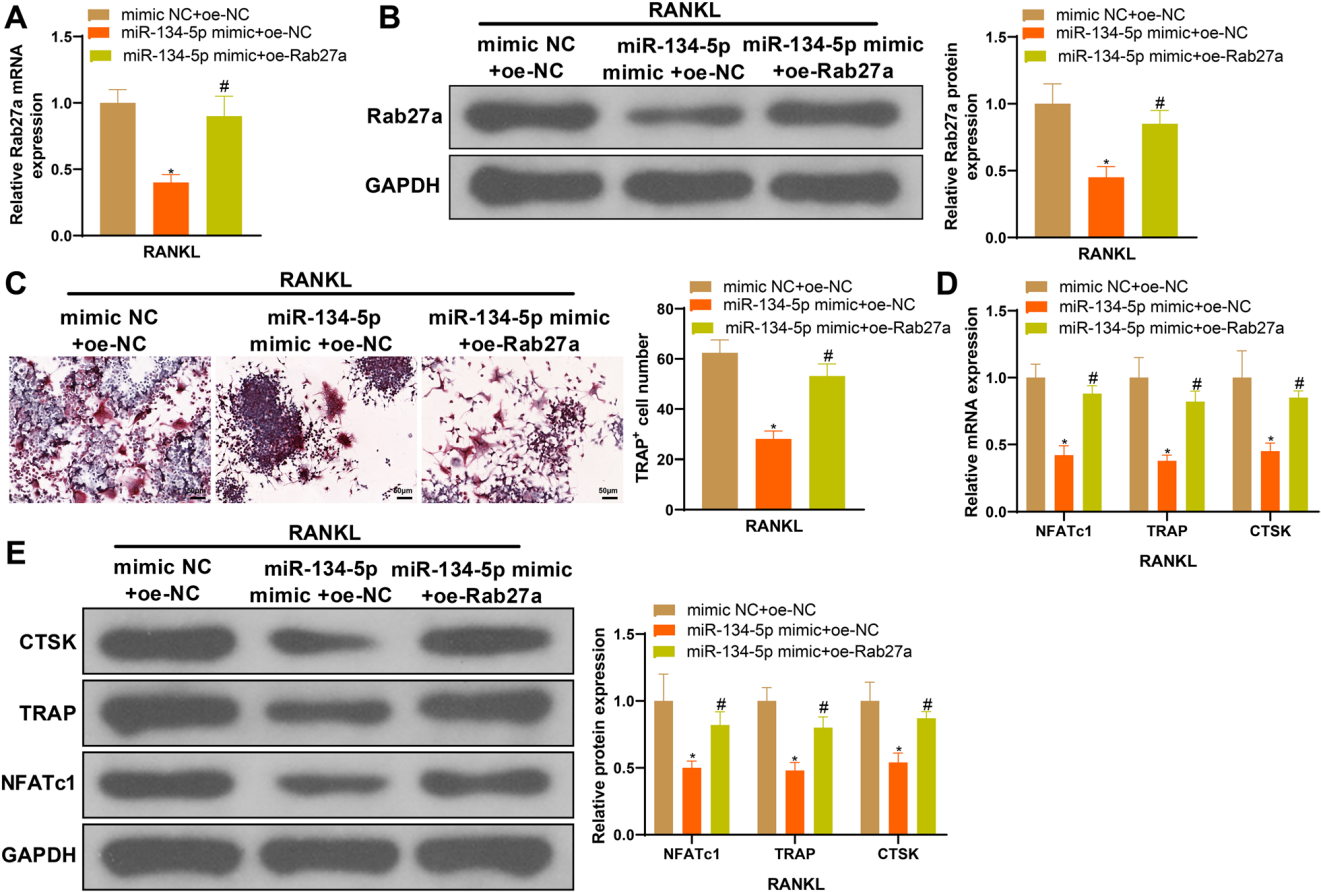
miR-134-5p negatively regulates Rab27a expression to mediate osteoclast differentiation. **A**, **B** Rab27a mRNA and protein expressions detected by RT-qPCR and western blot; **C** osteoclasts were observed after TRAP staining; **D**, **E** the biomarkers of osteoclasts, including NFATc1, TRAP and CTSK were detected using RT-qPCR and western blot. Data are expressed as mean ± standard deviation, cell experiments were repeated 3 times. *P < 0.05, when compared with mimic NC + oe-NC group; ^#^*P* < 0.05, when compared with miR-134-5p mimic + oe-NC group

The possible effects of miR-134-5p mimic alone and miR-134-5p mimic + oe-Rab27a on osteoclasts were assessed, and the data demonstrated that miR-134-5p mimic can suppress Rab27a expression and osteoclast formation compared with mimic NC, while such suppression could be reversed by further transfection with oe-Rab27a (Fig. 6A–E, ^*#*^*P* < 0.05). Collectively, these results suggest that miR-134-5p negatively regulates Rab27a expression to mediate osteoclast differentiation.

### Overexpression of Rab27a abolishes the effect of circ_0029463 knockdown on osteoclast differentiation

**Fig. 7:**
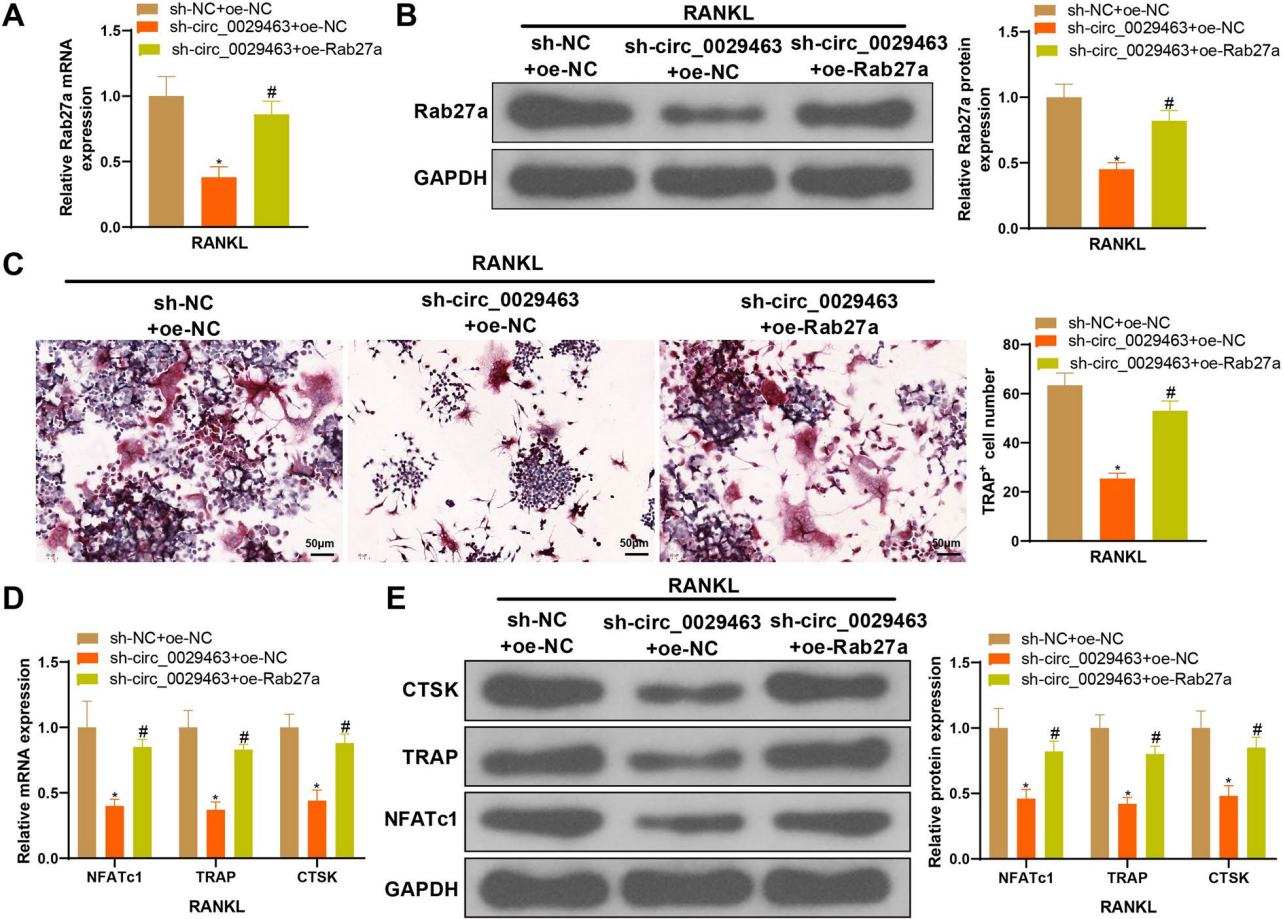
Overexpression of Rab27a abolish the effect of circ_0029463 knockdown on osteoclast differentiation. **A**, **B** Rab27a mRNA and protein expressions detected by RT-qPCR and western blot; **C** osteoclasts were observed after TRAP staining; **D**, **E** the biomarkers of osteoclasts, including NFATc1, TRAP and CTSK were detected using RT-qPCR and western blot. Data are expressed as mean ± standard deviation, cell experiments were repeated 3 times. *P < 0.05, when compared with mimic NC + oe-NC group; ^#^*P* < 0.05, when compared with sh-circ_0029463 + oe-NC group

Sh-circ_0029463 and oe-Rab27a were co-transfected with M-CSF + RANKL treated BMMs to verify the possible role of the circ_0029463/miR-134-5p/Rab27a axis in osteoclast formation. The detection of Rab27a expression showed that cells treated with sh-circ_0029463 showed suppressed Rab27a expression, whereas co-treatment with sh-circ_0029463 + oe-Rab27a elevated Rab27a expression compared to the negative control (Fig. 7A, B, ^*#*^*P* < 0.05). Evaluation of osteoclast formation revealed that the suppressive effect of sh-circ_0029463 on osteoclasts could be abolished by oe-Rab27a (Fig. 7C–E, ^*#*^*P* < 0.05), suggesting that Rab27a overexpression enhances osteoclast differentiation in BMMs.

## Discussion

Osteoblasts, which are essential for bone development, can be activated by multiple molecules that suppress or promote bone formation and/or bone resorption, resulting in skeletal disorders, including osteoporosis [24]. In this study, circ_0029463 was found to be upregulated in patients with osteoporosis and during osteoclastogenesis. circ_0029463 knockdown suppresses osteoblast differentiation and bone resorption. Furthermore, a circ_0029463-miR-134-5p-Rab27a network was identified in the regulation of osteoblast differentiation, contributing to an in-depth understanding of the molecular mechanisms underlying osteoporosis.

Notably, accumulating evidence demonstrates that circRNAs are involved in the regulation of age-associated pathologies such as cardio-cerebrovascular disorders, neurodegenerative disorders, tumors, diabetes, and osteoporosis [25]. As important epigenetic regulators, circRNAs are strongly linked to bone metabolic processes, including the differentiation of osteoblasts and osteoclasts [26]. For instance, circRNA_28313 knockdown attenuates osteoclast differentiation in BMMs [27]. Additionally, circ_0005564 silencing represses the differentiation of osteoblasts within bone marrow mesenchymal cells (BMSCs) [28] and circDOCK1 has been found to be a regulator of osteogenic sarcoma [29]. The first important finding of this study was that circ_0029463 was highly expressed in patients with osteoporosis. In vitro (BMMs) were stimulated with M-CSF and RANKL to mimic in vitro osteoclast formation. Osteoclasts differentiate from monocyte/macrophage lineage cells upon stimulation with M-CSF or RANKL. The RANKL/RANK pathway contributes to the activation of NFATc1, a major modulator of osteoclastogenesis, which induces the expression of osteoclastogenic markers [30]. Subsequently, functional experiments suggested that sh-circ_0029463 in BMMs led to a reduced number of newly formed osteoclasts and significantly attenuated bone resorption, corresponding to decreased expression of the osteoclast biomarkers NFATc1, TRAP, and CTSK. These results support the role of circ_0029463 in osteoclast differentiation. However, the effect of circ_0029463 on osteoclast differentiation remained unclear.

Our study explored the downstream mechanism of circ_0029463 in osteoclast differentiation and found that circ_0029463 binds to miR-134-5p. Moreover, inhibition of miR-134-5p reversed the inhibitory effects of sh-circ_0029463 on osteoclast differentiation. miR-134 has been shown to inhibit the chondrogenic differentiation of BMSCs [31]. A more recent study demonstrated that inhibition of miR-134-5p can expedite osteoclast formation and cell proliferation while suppressing cell apoptosis [20], which is consistent with our findings. A previous study identified decreased miR-134-5p during osteoclast formation in ovariectomized (OVX) mouse models, and the same study also found that overexpression of miR-134-5p enhanced the osteoclast formation [20]. Among multiple regulatory factors, miRNAs have been identified as valid tools for their regulation on osteoclast formation and bone resorption. In a previous study, several transcripts were predicted to be targets of miR-134-5p, including Rab27a and Itgb1; these transcripts were also found to be involved in osteoclastic progression [20, 32]. More recently, the relationship between miR-134-3p and Rab27a was validated in human ovarian cancer stem cells [22]. Furthermore, RIP and dual-luciferase reporter gene assays revealed a binding relationship between miR-134-3p and Rab27a. Detection of cellular biological functions identified that miR-134-5p could suppress the osteoblast differentiation by targeting Rab27a. Rab27a and Rab27b affect stimulation-dependent RANKL secretion by secretory lysosomes in osteoblasts [33]. Moreover, Rab27a expression increases during osteoclast differentiation and its deficiency leads to reduced osteoclast activation [34]. The involvement of Rab27a in osteoclast differentiation was also observed in OVX mouse models, which showed Rab27a was restrained by miR-124 to attenuate osteoclastogenesis [35, 36]. These results clarified the role of Rab27a in osteoclast differentiation and hinted at the complications of osteoclast differentiation. Our experiments further showed that Rab27a overexpression in the presence of miR-134-5p mimic or sh-circ_0029463 upregulated the expression of NFATc1, TRAP, and CTSK and enhanced osteoblast formation and bone resorption, suggesting the involvement of the circ_0029463-miR-134-5p-Rab27a network in osteoblast formation.

This study has some limitations that should be mentioned when interpreting the results. First, osteoblast differentiation may be the result of interactions and communication between multiple cellular agents or pathways; however, this study only explored the involvement of circ_0029463-miR-134-5p-Rab27a in osteoblast formation. Further studies are required to establish a comprehensive understanding of osteoblast differentiation. In addition to that, multiple evidences demonstrated that circRNAs exerts their function through being transported by extracellular vesicles [37, 38]. Future studies are required to validate whether circ_0029463 in extracellular vesicles can still promote osteoblast differentiation.

Collectively, the present study demonstrates that circ_0029463 functions as a ceRNA of miR-134-5p to suppress miR-134-5p expression, which can release Rab27a, thereby promoting osteoblast differentiation. This study highlights the role of the circ_0029463-miR-134-5p-Rab27a network in osteoblast formation and contributes to a better understanding of the pathology of osteoblast differentiation. The results of this study may provide a theoretical basis for targeting key molecules, such as circ_0029463, as a promising approach for anti-osteoporosis interventions. However, further studies and multiple tests are required for validation before clinical translation.

## Data Availability

The datasets used or analyzed during the current study are available from the corresponding author on reasonable request.
